# Supplementary figures and images for: Bayesian accounts of perceptual decisions in the nonclinical continuum of psychosis: Greater imprecision in both top-down and bottom-up processes
Source: PLoS Comput Biol. 2023 Nov 21;19(11):e1011670. doi: 10.1371/journal.pcbi.1011670 (PMC10697609; doi:10.1371/journal.pcbi.1011670)

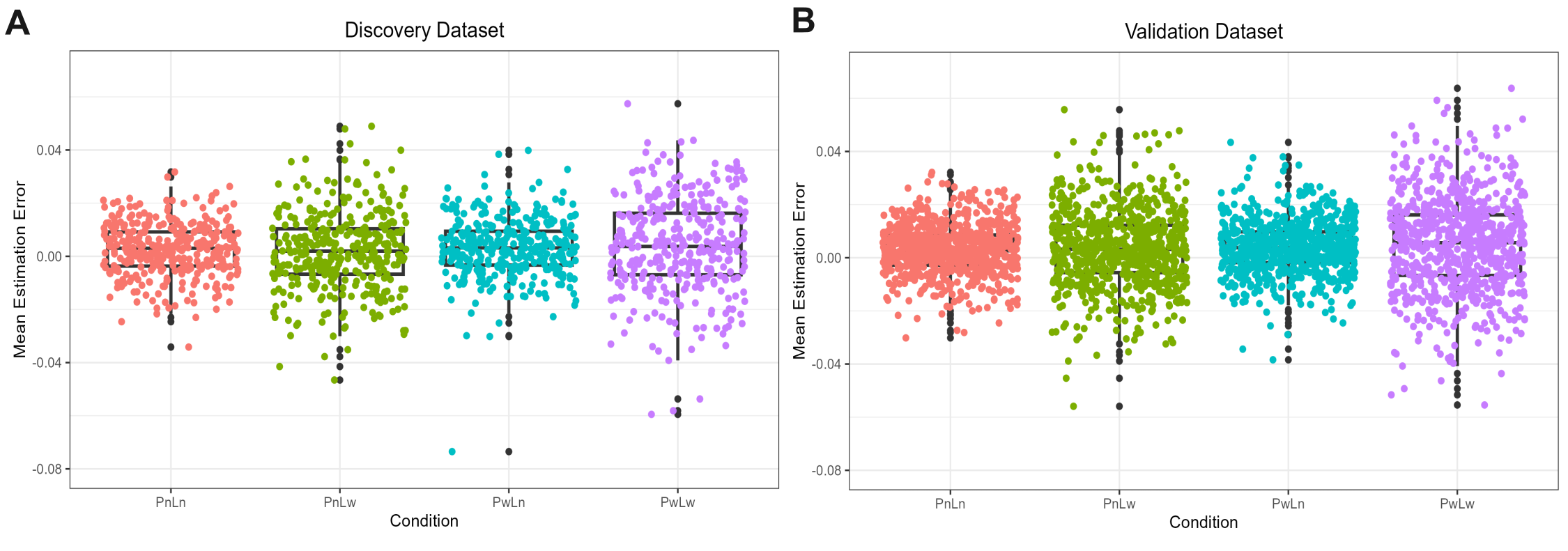

Supplement: S1 Fig — Comparison of mean estimation error performance across trial conditions in A) the discovery and B) the validation datasets. Demonstrating the variability in mean estimation errors across each of the four the trial conditions to supplement task performance analyses. Conditions: PnLn = narrow prior, narrow likelihood (red dots); PnLw = narrow prior, wide likelihood (green dots); PwLn = wide prior, narrow likelihood (teal dots); PwLw = wide prior, wide likelihood (purple dots). (TIFF) [file pcbi.1011670.s001.tiff]

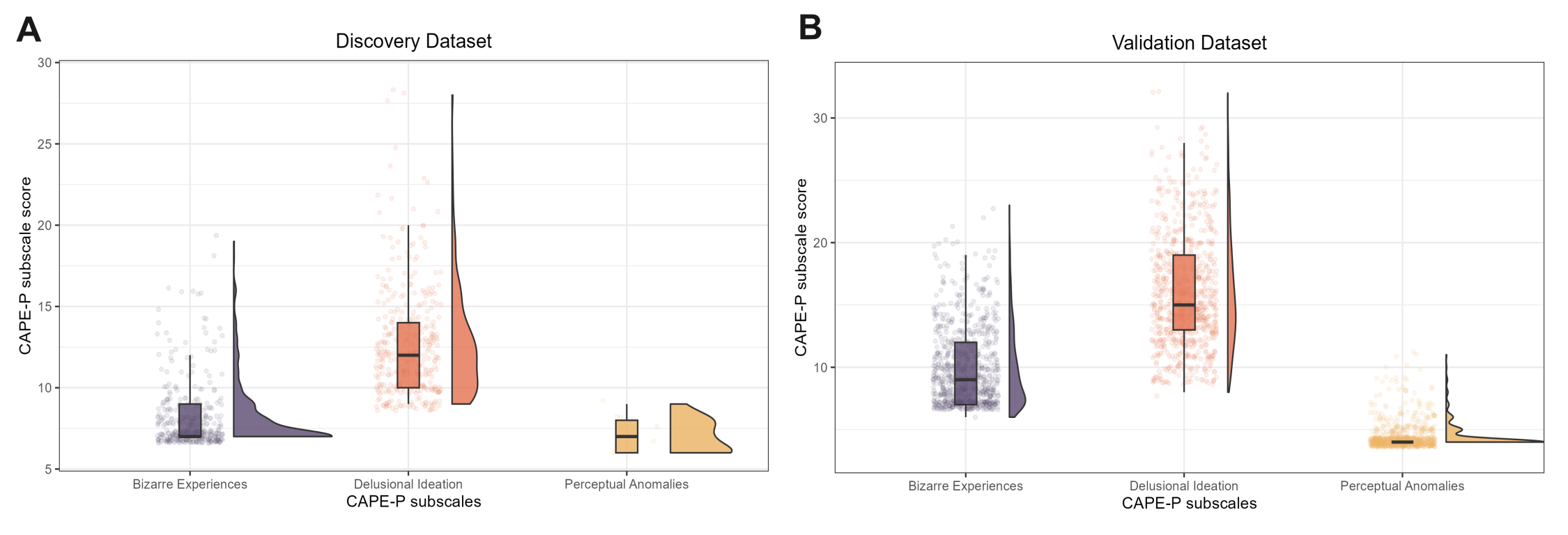

Supplement: S2 Fig — Distribution of CAPE-P subscale scores (bizarre experiences, delusional ideation and perceptual anomalies) in A) the discovery dataset (n = 363) and B) validation dataset (n = 782). (TIFF) [file pcbi.1011670.s002.tiff]
