# Supplementary material for: Bayesian accounts of perceptual decisions in the nonclinical continuum of psychosis: Greater imprecision in both top-down and bottom-up processes
Source: PLoS Comput Biol. 2023 Nov 21;19(11):e1011670. doi: 10.1371/journal.pcbi.1011670 (PMC10697609; doi:10.1371/journal.pcbi.1011670)
Supplement: S1 Table — (PDF) [file pcbi.1011670.s003.pdf]

	Discovery dataset			Validation dataset		
	Median	Optimal	p-value	Median	Optimal	p-value
PnLn	0.638	0.465	$<2.2 \times 10^{-16}$	0.622	0.465	$<2.2 \times 10^{-16}$
PnLw	0.517	0.122	$<2.2 \times 10^{-16}$	0.516	0.122	$<2.2 \times 10^{-16}$
PwLn	0.828	0.909	$<2.2 \times 10^{-16}$	0.834	0.909	$<2.2 \times 10^{-16}$
PwLw	0.706	0.616	$<2.2 \times 10^{-16}$	0.725	0.616	$<2.2 \times 10^{-16}$
