# Supplementary material for: Bayesian accounts of perceptual decisions in the nonclinical continuum of psychosis: Greater imprecision in both top-down and bottom-up processes
Source: PLoS Comput Biol. 2023 Nov 21;19(11):e1011670. doi: 10.1371/journal.pcbi.1011670 (PMC10697609; doi:10.1371/journal.pcbi.1011670)
Supplement: S2 Table — (PDF) [file pcbi.1011670.s004.pdf]

	Discovery dataset			Validation dataset		
	Median	Optimal	p-value	Median	Optimal	p-value
PnLn	0.00105	0.0016	0.178	9.5×10^{-4}	0.0016	0.0311
PnLw	0.00239	0.0016	6.64×10^{-15}	2.4×10^{-3}	0.0016	$<2.2 \times 10^{-16}$
PwLn	2.91×10^{-3}	0.0185	$<2.2 \times 10^{-16}$	3.1×10^{-3}	0.0185	$<2.2 \times 10^{-16}$
PwLw	0.00623	0.0185	6.77×10^{-16}	6.4×10^{-3}	0.0185	$<2.2 \times 10^{-16}$
