# Supplementary material for: Bayesian accounts of perceptual decisions in the nonclinical continuum of psychosis: Greater imprecision in both top-down and bottom-up processes
Source: PLoS Comput Biol. 2023 Nov 21;19(11):e1011670. doi: 10.1371/journal.pcbi.1011670 (PMC10697609; doi:10.1371/journal.pcbi.1011670)
Supplement: S3 Table — In the manuscript, correlation analyses compare the associations between psychotic-like experiences with (1) sensory weight, (2) variability in sensory weight, (3) subjective likelihood variance (although this is calculated from a separate task) and (4) subjective prior variance. Given this, four comparisons are (at most) being made across the analyses. Adjusted p-values for these comparisons are demonstrated here, along with bootstrapped confidence intervals with 1000 bootstrapped replicates. (PDF) [file pcbi.1011670.s005.pdf]

Variables	Dataset	Correlation	Bonferroni correction	Bootstrapped 95% CI
Log(CAPE-P) and sensory weight	Discovery<br>Validation	r = 0.17, p = .0285<br>r = 0.08, p = .0242	p_{adj} = .114<br>p_{adj} = .0970	[0.015, 0.221]<br>[0.0052, 0.1568]
CAPE-P and subjective likelihood variance	Discovery<br>Validation	r = 0.08, p = .156<br>r = 0.15, p = 1.5x10^{-5}	p_{adj} = .627<br>p_{adj} = 9.8x10^{-5}	[-0.031, 0.182]<br>[0.0838, 0.2244]
CAPE-P and subjective prior variance	Discovery<br>Validation	r = 0.11, p = .0458<br>r = 0.14, p = 1.1x10^{-4}	p_{adj} = .183<br>p_{adj} = 4.6x10^{-4}	[0.0002, 0.214]<br>[0.069, 0.219]
