# Supplementary material for: Bayesian accounts of perceptual decisions in the nonclinical continuum of psychosis: Greater imprecision in both top-down and bottom-up processes
Source: PLoS Comput Biol. 2023 Nov 21;19(11):e1011670. doi: 10.1371/journal.pcbi.1011670 (PMC10697609; doi:10.1371/journal.pcbi.1011670)
Supplement: S4 Table — (PDF) [file pcbi.1011670.s006.pdf]

Discovery dataset (n = 363)				
**Variable**	**CAPE-P subscale**	**Correlation**	**Bonferroni correction (n=9)**	**Bootstrapped 95% CI**
Sensory weight	Bizarre experiences	r=0.06, p=.260	p=.999	[-0.05, 0.17]
	Delusional ideation	r=0.11, p=.039	p=.349	[0.002, 0.22]
	Perceptual anomalies	r=0.04, p=.500	p=.999	[-0.063, 0.14]
Subjective likelihood variance	Bizarre experiences	r=0.07, p=.180	p=.999	[-0.03, 0.17]
	Delusional ideation	r=0.09, p=.100	p=.911	[-0.02, 0.19]
	Perceptual anomalies	r=0.045, p=.410	p=.999	[-0.06, 0.15]
Subjective prior variance	Bizarre experiences	r=0.078, p=.160	p=.999	[-0.032, 0.18]
	Delusional ideation	r=0.12, p=.024	p=.215	[0.023, 0.226]
	Perceptual anomalies	r=0.04, p=.48	p=.999	[-0.067, 0.145]
