# Supplementary material for: Bayesian accounts of perceptual decisions in the nonclinical continuum of psychosis: Greater imprecision in both top-down and bottom-up processes
Source: PLoS Comput Biol. 2023 Nov 21;19(11):e1011670. doi: 10.1371/journal.pcbi.1011670 (PMC10697609; doi:10.1371/journal.pcbi.1011670)
Supplement: S5 Table — (PDF) [file pcbi.1011670.s007.pdf]

Validation dataset (*n* = 782)				
**Variable**	**CAPE-P subscale**	**Correlation**	**Bonferroni correction (n=9)**	**Bootstrapped 95% CI**
Sensory weight	Bizarre experiences	*r*=0.11, *p*=.0009	*p*=.008	[0.51, 0.19]
	Delusional ideation	*r*=0.06, *p*=.11	*p*=.996	[-0.01, 0.12]
	Perceptual anomalies	*r*=0.07, *p*=.056	*p*=.505	[-0.004, 0.14]
Subjective likelihood variance	Bizarre experiences	*r*=0.13, *p*=.0002	*p*=.001	[0.07, 0.21]
	Delusional ideation	*r*=0.14, *p*=.0001	*p*=.001	[0.07, 0.22]
	Perceptual anomalies	*r*=0.13, *p*=.0005	*p*=.005	[0.56, 0.19]
Subjective prior variance	Bizarre experiences	*r*=0.16, p=1.4x10^{-5}	*p*=.0001	[0.09, 0.23]
	Delusional ideation	*r*=0.11, *p*=.002	*p*=.018	[0.04, 0.18]
	Perceptual anomalies	*r*=0.11, *p*=.003	*p*=.027	[0.04, 0.18]
