# Supplementary material for: Bayesian accounts of perceptual decisions in the nonclinical continuum of psychosis: Greater imprecision in both top-down and bottom-up processes
Source: PLoS Comput Biol. 2023 Nov 21;19(11):e1011670. doi: 10.1371/journal.pcbi.1011670 (PMC10697609; doi:10.1371/journal.pcbi.1011670)
Supplement: Attachment — Submitted filename: Response to reviewers.docx [file pcbi.1011670.s008.docx]

Response to reviewers

Plos Computational Biology

**Title: Bayesian Accounts of Perceptual Decisions in the Nonclinical Continuum of Psychosis: Greater Imprecision in Both Top-down and Bottom-up Processes**

*Reviewer comments in blue italics*, author comments in normal font, **changes to manuscript in bold**

# **Reviewer #1**

## General comment

*In this study the authors used an online psychophsyical experiment to investigate the neurocomputational mechanisms underlying psychosis proneness in two large samples of healthy participants, employing a task designed to test prior and likelihood estimates independently. The main result, which is found consistently in both samples, is an increased weighting of the likelihood relative to prior information in relation to psychosis-proneness. In addition, psychosis-proneness is reported to be associated with reduced perceived task stability as reflected by both subjective likelihood variance and subjective prior variance. The authors interpret their findings as evidence for both weaker bottom-up and top-down perceptual processes that leads to an overweighting of sensory evidence relative to prior information.*

*The study is timely and tackles an important question in the context of predictive processing accounts of psychosis. The task design and experimental methods are sound and the use of independent discovery and confirmation samples lends particular strength to the findings. So overall, there is much to like about this study. The only major concerns I have relate to how the measures of prior and likelihood precision are derived and whether the conclusions that are based on these measures are tenable. As it stands, I’m therefore not sure whether the novel part of the findings, relating to prior precision, is supported by the data.*

## Specific points

*1. I have to admit that I struggled to understand the rationale for (and computation of) subjective task variance. This is a critical measure, because much of the author’s interpretation of the findings is based on it. But how can subjective prior variance be derived from actual likelihood variance and optimal sensory weights? This really needs to be unpacked, so the reader can understand better what the measure of subjective prior variance really means and how it should be interpreted.*

- We acknowledge that the explanation of subjective prior variance could be clearer. We have included some additional explanation in the description of subjective prior variance in the manuscript (pg 12): **Subjective prior variance (**$\boldsymbol{\sigma}_{\boldsymbol{P}}^{\boldsymbol{2}}$**)** **is an estimate of participants’ subjective representation of the variability in prior information (i.e., the variability of the thrower). This is expected to change between blocks depending on which thrower is throwing the coin (as one is more variable than the other) and can be used as a proxy to determine participants’ individual estimates of how much variability or overall uncertainty there is in prior information. Subjective prior variance is calculated as a weighted combination of subjective likelihood variance and sensory weight, which is derived by rearranging equation 2 as follows:**

$$\sigma_{P}^{2}= \frac{\sigma_{SL}^{2}*sw}{(1-sw)}$$

**Higher values for the subjective prior variance should be interpreted as more subjective uncertainty (i.e., increased variability) in the internal representations of prior information.**

*2. Relatedly, the authors conclude that “that the perceived instability in prior information is driving a greater reliance on likelihood information throughout the task”. This interpretation is, in my opinion, not tenable. How do we know that this is the direction of causality? Is it indeed perceived instability of priors that drives overreliance? Or may it be the other way around, i.e., could a primary overreliance on sensory information (e.g. due to a heightened dopamine availability, as discussed by the authors) lead to the experience of aberrant salience and consequently to reduced subjective task stability? Whether the latter scenario is a likely one or not, the causal conclusion that subjective prior variance drives overreliance on sensory evidence does not seem to be supported by the data.*

- Thank you for raising this question. We now clarify this point in the manuscript (pg 22): **We also found that psychotic-like experiences positively correlated with both subjective likelihood variance *and* subjective prior variance in the larger, validation dataset, insinuating the perception of generally greater task instability. We argue that the heightened reliance on likelihood information throughout the task appears to be driven by noisier representations of prior information (Fig 6), rather than by the sharpening of sensory representations, which were also shown to be noisier as psychotic-like experiences increased (Fig 5). Alternatively, the heightened reliance on likelihood information could lead to the experience of aberrant salience, which could be more generally influencing the perception of greater task instability.**

*3. Again in relation to the measures of perceived variance: It is laudable that the task modulates uncertainties in likelihood and prior information independently, but while there is clear evidence for an overreliance on sensory information, no direct measure is available for the reliance on prior information. Instead, a measure of subjective variance is used. It is unclear however, whether this subjective task instability indeed reflects reduced prior precision. To my mind, this is only one possible interpretation. Couldn’t increased subjective prior variance also be a result of overreliance on sensory information (see above)? Wouldn’t it be conceivable that the prior variance is perceived as high, but that this does not directly affect the estimation of prior precision in each trial, i.e. in the actual inferences made? I would like the authors to provide a more balanced discussion of this issue and to tone down the interpretation of increased subjective prior variance as directly reflecting reduced prior precision.*

- Upon reflection, we agree that participants’ general overestimation of uncertainty could be a result of the overreliance on sensory information. We have amended the discussion to include this interpretation and reduce the language inferring causality, such as:
  - **Although this cannot be directly inferred…**
  - **…substantiates our hypothesis** (rather than “substantiates our findings”)
  - **Despite this, the increased subjective prior variance could also be a result of an overreliance on sensory observations, rather than a driver for it, which cannot be decisively ambiguated from our findings.**
  - In the description of Figure 7… “where the decrease in prior precision is **hypothesized to be** driving a shift in the posterior towards likelihood information”
- We also clarify the meaning of the sensory weight, which is indeed a measure of reliance on prior information (pg 10):
  - **We use the term sensory weight throughout the manuscript. Note, however, that the sensory weight is a relative measure for how much people rely on sensory information versus prior information, hence it is simultaneously a measure of “*reliance on sensory information*” and “*reliance on prior information*”.**

*4. I found parts of the introduction a bit difficult to follow:*

*“…discrepancies within the literature describe both increases and decreases in the relative weighting of likelihood to prior information” (lines 94 ff.). These discrepancies do not follow from what is said in the two sentences before, but rather from what is said in the preceding paragraph.*

- Thank you for pointing this out. We have edited this sentence to aid clarity (pg 4): **discrepancies within the literature describe alternative accounts of how precision is afforded to likelihood and prior information.**

*“…to determine whether an overreliance on prior or likelihood information is driven by more precise priors or more precise likelihoods” (lines 116 f.). This is a muddled sentence that needs to be unpacked. How can “prior or likelihood information” be driven by “precise priors of ore precise likelihood”?*

- Thank you for highlighting this sentence too. We have unpacked this into a more detailed description (pg 5): **Our approach enabled us to determine whether an overreliance on prior or likelihood information correlates with psychotic-like experiences. It also allowed us to elucidate whether the subjective uncertainty associated with the representation of likelihood and/or prior information (a proxy for the precision afforded to each source of information) was associated with psychotic-like experiences.**

*5. Task instructions: Did the participants know, as in were explicitly told, which block they were in? Was the order of blocks randomized or alternating (aiding the participant’s knowledge regarding block structure). Or did they have to infer the thrower (and hence the prior) from the distribution of throws?*

- Thank you for this comment, this is important information which could be clearer. This information is alluded to in the section outlining task instructions:
  - “Participants were also informed that the person throwing the coin changed between blocks, and one thrower was more accurate than the other. They were told that both throwers aimed at the screen centre (indicating the mean of the prior). Although they were not explicitly told which thrower was better or worse, this could be inferred through the distribution of previous coin locations from trial-to-trial.”
- We have also added this sentence to further aid clarity (pg 7): **Although participants were not explicitly told which block they were in, they were informed that thrower A and thrower B alternated between blocks. This was randomised across participants so that half of participants received thrower A first, and half the participants received thrower B first. Thus, participants could infer the prior information (regarding the variability of the thrower) based on the distribution of throws.**

*6. Regarding the authors’ suggestion for future studies to look at separate measures for delusion and hallucination proneness (lines 575 ff.): They may refer to a recently published study that did this and found evidence for a reduced reliance on priors relative to sensory evidence in relation to both delusion and hallucination proneness, using perceptual decision making tasks (*[*https://pubmed.ncbi.nlm.nih.gov/36440751/*](https://pubmed.ncbi.nlm.nih.gov/36440751/)*).*

- Thank you for pointing this out this exciting paper, which was published after our submission to Plos Computational Biology. We agree that it is relevant to our findings, and thus has been included in the final paragraph of the discussion (pg 28): **A recent study found evidence for a reduced reliance on priors to sensory evidence in relation to both delusion *and* hallucination proneness [49], which is consistent with our data and proposed model for the continuum of psychosis (Fig 7).**

# **Reviewer #2**

## General comment

*I read and enjoyed Goodwin and colleagues report of their experiment on the use of likelihoods and priors in perceptual decision-making in the general population and how they relate to psychosis-like experiences. They report that sensory inputs are overweighted in a manner that correlates with CAPE-P scores. This is a potentially useful contribution to a growing empirical literature. However, I have some substantive to concerns which I would like the reviewers to address with new analyses.*

## Specific points

*First, there is a clear multiple comparisons problem, and the effects they report are quite small - when appropriate correction for multiple correlations is applied, I suspect many (at least in the discovery sample) would not survive.*

- Thank you for this comment, we appreciate this concern. We have incorporated Table S3 into the supplementary information of our manuscript which we preface in the statistical analysis section (pg 13): **Although we believe that accounting for multiple comparisons with Bonferroni corrections is too conservative of an approach for our research design and may increase the risk of a type 2 error [25], we have included adjusted p-values for our main correlations of interest as a comparison to bootstrapped confidence intervals in supplementary Table S3.**
- To explain this further, in the discussion section (pg 27) we now say: **Small effect sizes are common in the literature regarding psychotic-like experiences as a trait-like characteristic, with these effects naturally larger in clinical populations. Despite this, the reported subclinical effects are modest but significant and robustly replicated across both datasets. We argue that multiple comparisons such as Bonferroni corrections are too conservative to be applied to this data given that some of the measures are correlated and others determined from independent datasets. Given that four comparisons (at most) are being made between psychotic-like experiences and parameters of interest across the analyses, adjusted p-values with Bonferroni corrections can be seen in Table S3. This shows that the positive relationship between psychotic-like experiences and subjective likelihood variance, as well as subjective prior variance, remain significant in the larger, validation dataset with these corrections applied. Instead of this approach, we have incorporating bootstrapped confidence intervals (with 1000 bootstrapped replicates) into our analyses (also shown in Table S3), which we use for our interpretations.**

***Table S3. Adjusted p-values with Bonferroni corrections for multiple comparisons applied to main correlations of interest***

| **Variables** | **Dataset** | **Correlation** | **Bonferroni correction** | **Bootstrapped 95% CI** |
| --- | --- | --- | --- | --- |
| **Log(CAPE-P) and sensory weight** | **Discovery** | ***r* = 0.17, *p* = .0285** | ***p*_adj_ = .114** | **[0.015, 0.221]** |
|  | **Validation** | ***r* = 0.08, *p* = .0242** | ***p*_adj_ = .0970** | **[0.0052, 0.1568]** |
| **CAPE-P and subjective likelihood variance** | **Discovery** | ***r* = 0.08, *p* = .156** | ***p*_adj_ = .627** | **[-0.031, 0.182]** |
|  | **Validation** | ***r* = 0.15, *p* = 1.5x10^-5^** | ***p*_adj_ = 9.8x10^-5^** | **[0.0838, 0.2244]** |
| **CAPE-P and subjective prior variance** | **Discovery** | ***r* = 0.11, *p* = .0458** | ***p*_adj_ = .183** | **[0.0002, 0.214]** |
|  | **Validation** | ***r* = 0.14, *p* = 1.1x10^-4^** | ***p*_adj_ = 4.6x10^-4^** | **[0.069, 0.219]** |

***Note. In the manuscript, correlation analyses compare the associations between psychotic-like experiences with (1) sensory weight, (2) variability in sensory weight, (3) subjective likelihood variance (although this is calculated from a separate task) and (4) subjective prior variance. Given this, four comparisons are (at most) being made across the analyses. Adjusted p-values for these comparisons are demonstrated here, along with bootstrapped confidence intervals with 1000 bootstrapped replicates.***

*More importantly, I think there is some misunderstanding about the different kinds of Prior overweighting theories. There are some that suggest strong priors would induce aberrant prediction errors - which then relate to delusions (though this is not a commonly espoused theory). Instead, prior overweighting (due to under-precise inputs or over-precise priors) is more commonly associated with hallucinations. Did any of these participants actually have hallucinations? In the absence of hallcuinations, we might not expect prior overweighting.*

- Indeed, our participants did not have clinical hallucinations as they were sampled from a non-clinical, healthy population, and hence this could explain the lack of prior overweighting. This is illustrated in the low distribution of scores for the CAPE-P subscale item ‘perceptual anomalies’ in Figure 1 below. Instead, our analyses utilise the CAPE-P score, which measures participants psychotic-like positive experiences more generally (rather than specifically delusions, hallucinations etc). While it is true that prior overweighting theories are more commonly associated with hallucinations, the interpretation of our data suggests that increased psychotic-like experiences are positively associated with an overreliance on likelihood information. Thus, this renders the discussion regarding prior overweighting theories less relevant. Instead, we discuss whether this overreliance on likelihood information could be driven by increased precision in likelihood information or decreased precision in prior information (which, based on our data, we suggest being the latter). For clarification, we now say (pg 28):
- “…hallucination formation has contrastingly been characterised as abnormally strong priors, leading to the formation of false percepts [10].” **Although our data is not consistent with the overweighting of priors account, our participants did not experience actual hallucinations as they were sampled from a non-clinical population, hence we cannot completely rule out this account in hallucinating individuals.**

*One way to address these issues with the data in hand: it would be helpful to know what the paranoia, bizarre ideas, and perceptual aberrations subscake scores were, and whether, people with more perceptual aberrations overweighted, and whether the paranoid participants had stringer volatility beliefs (as has been shown repeatedly by other labs). Perhaps the authors could pre-register some predictions about how specific symptoms might relate to behavior in their task before analyzing the subscale scores?*

- We appreciate the suggestion to investigate how the suggested subscales relate to task behaviour. We demonstrate the distribution of these subscales in both the discovery and validation datasets below, and apply the suggested analyses, accounting for 9 comparisons of interest in Tables S4 and S5 (which we include in supplementary material). We now say (pg 28):
- **We followed up these findings in the current study to determine whether there was a similar relationship in CAPE-P subscale scores, namely bizarre experiences, delusional ideation, and perceptual anomalies [50] (see supplementary Fig. S1 for a distribution of the subscales in our data). We found that delusional ideation was positively correlated with subjective prior variance in both discovery and validation sets (see supplementary Table S4 and S5). We also found other significant, yet unspecific, relationships although only for the validation set. However, one should be cautious when interpreting these findings since these subscales are based on limited number of items (bizarre experiences are defined by 7 items, delusional ideation is defined by 9 items, and perceptual anomalies are defined by 4 items [50]). Hence, we turn to the interpretation of the 20 item CAPE-P instead and suggest future research to employ separate measures to compare hallucinatory and delusional-like experiences…**
- Our concern lies with inferring strong claims based on shallow data (i.e., few items for each score), which is why we believe our interpretations should be based on CAPE-P scores which are defined by 20 items, rather than the subscale scores as suggested. Instead, in the final paragraph of the discussion we propose that future research should replicate the experimental paradigm, including separate measures of hallucinatory and delusion-like experiences. To make this clearer in the manuscript, we have included examples of questionnaires that could be employed in future research (pg 29): **Examples of specific measures include Peters Delusion Inventory [51] and Cardiff Anomalous Perceptions Scale [52] which are designed to measure delusional ideation and hallucinatory experiences in the general population respectively.**

***Figure S1.* Distribution of CAPE-P subscale scores (bizarre experiences, delusional ideation and perceptual anomalies) in A) the discovery dataset (*n*=363) and B) validation dataset (*n*=782)**

***Table S4.* Spearman correlation between CAPE-P subscale scores (bizarre experiences, delusional ideation and perceptual anomalies) and variables of interest (sensory weight, subjective likelihood variance and subjective prior variance) in the discovery dataset (*n*=363)**

| **Discovery dataset (*n* = 363)** | | | | |
| --- | --- | --- | --- | --- |
| **Variable** | **CAPE-P subscale** | **Correlation** | **Bonferroni correction (n=9)** | **Bootstrapped 95% CI** |
| **Sensory weight** | **Bizarre experiences** | ***r*=0.06, *p*=.260** | ***p*=.999** | **[-0.05, 0.17]** |
|  | **Delusional ideation** | ***r*=0.11, *p*=.039** | ***p*=.349** | **[0.002, 0.22]** |
|  | **Perceptual anomalies** | ***r*=0.04, *p*=.500** | ***p*=.999** | **[-0.063, 0.14]** |
| **Subjective likelihood variance** | **Bizarre experiences** | ***r*=0.07, *p*=.180** | ***p*=.999** | **[-0.03, 0.17]** |
|  | **Delusional ideation** | ***r*=0.09, *p*=.100** | ***p*=.911** | **[-0.02, 0.19]** |
|  | **Perceptual anomalies** | ***r*=0.045, *p*=.410** | ***p*=.999** | **[-0.06, 0.15]** |
| **Subjective prior variance** | **Bizarre experiences** | ***r*=0.078, *p*=.160** | ***p*=.999** | **[-0.032, 0.18]** |
|  | **Delusional ideation** | ***r*=0.12, *p*=.024** | ***p*=.215** | **[0.023, 0.226]** |
|  | **Perceptual anomalies** | ***r*=0.04, *p*=.48** | ***p*=.999** | **[-0.067, 0.145]** |

***Table S5.* Spearman correlation between CAPE-P subscale scores (bizarre experiences, delusional ideation and perceptual anomalies) and variables of interest (sensory weight, subjective likelihood variance and subjective prior variance) in the validation dataset (*n*=782)**

| **Validation dataset (*n* = 782)** | | | | |
| --- | --- | --- | --- | --- |
| **Variable** | **CAPE-P subscale** | **Correlation** | **Bonferroni correction (n=9)** | **Bootstrapped 95% CI** |
| **Sensory weight** | **Bizarre experiences** | ***r*=0.11, *p*=.0009** | ***p*=.008** | **[0.51, 0.19]** |
|  | **Delusional ideation** | ***r*=0.06, *p*=.11** | ***p*=.996** | **[-0.01, 0.12]** |
|  | **Perceptual anomalies** | ***r*=0.07, *p*=.056** | ***p*=.505** | **[-0.004, 0.14]** |
| **Subjective likelihood variance** | **Bizarre experiences** | ***r*=0.13, *p*=.0002** | ***p*=.001** | **[0.07, 0.21]** |
|  | **Delusional ideation** | ***r*=0.14, *p*=.0001** | ***p*=.001** | **[0.07, 0.22]** |
|  | **Perceptual anomalies** | ***r*=0.13, *p*=.0005** | ***p*=.005** | **[0.56, 0.19]** |
| **Subjective prior variance** | **Bizarre experiences** | ***r*=0.16, *p*=1.4x10^-5^** | ***p*=.0001** | **[0.09, 0.23]** |
|  | **Delusional ideation** | ***r*=0.11, *p*=.002** | ***p*=.018** | **[0.04, 0.18]** |
|  | **Perceptual anomalies** | ***r*=0.11, *p*=.003** | ***p*=.027** | **[0.04, 0.18]** |
