# Supplementary material for: Bayesian accounts of perceptual decisions in the nonclinical continuum of psychosis: Greater imprecision in both top-down and bottom-up processes
Source: PLoS Comput Biol. 2023 Nov 21;19(11):e1011670. doi: 10.1371/journal.pcbi.1011670 (PMC10697609; doi:10.1371/journal.pcbi.1011670)
Supplement: Attachment — Submitted filename: Response to reviewers.docx [file pcbi.1011670.s009.docx]

Dear Professor Mathys,

Thank you for giving us the opportunity to submit our revised manuscript “Bayesian accounts of perceptual decisions in the nonclinical continuum of psychosis: Greater imprecision in both top-down and bottom-up processes” for publication in PLOS Computational Biology. We appreciate the time and effort the reviewers dedicated to providing insightful feedback and valuable improvements to our paper. We have modified the manuscript according to the review recommendations. Please see below for *reviewer comments in blue italics*, author comments in normal font and **changes to the manuscript in bold**. These changes are also highlighted in the tracked changes version of the manuscript file. All page numbers refer to the revised/tracked changes manuscript file, and we have also uploaded a clean version of the revised manuscript.

# **Reviewer #1**

*I would like to thank the authors for fully addressing all my concerns. I am looking forward to seeing this excellent paper published.*

Thank you very much for this comment.

# **Reviewer #3**

*In this study, across two population datasets, psychotic-like experiences (positive symptoms in the CAPE) were associated with: weighting of sensory information, loss of subjective prior precision, loss of subjective likelihood precision (in the validation study only), (no relation to 'prior sensitivity', i.e. changes in sensory weight from block to block as prior precision changes)*

*I found this paper interesting and a useful contribution, given it is rare that people design experiments to manipulate prior and likelihood precision at the same time in the schizophrenia spectrum, yet very important to do so. I thought the authors' responses to the other reviewers concerns were valid. The use of two large samples is reassuring: the effects are quite small but the replication sample assuages any multiple comparison concerns that I might have had.*

Thank you for the positive comments on our work.

*I don't have suggestions for major revisions but there are a few points that I would like to be addressed:*

*p15 - I am perhaps misunderstanding something but why does the likelihood-only task show large effects of likelihood precision on estimation errors, but the main task doesn't show these effects or effects of prior precision (except in the validation dataset)?*

Thank you for this comment. In the likelihood-only task, we expect participants to make greater estimation errors in the wide likelihood condition, as there is more uncertainty associated with the true coins position as compared to the narrow likelihood condition. This is highlighted in the results section (pg 15): “likelihood uncertainty manipulation functioned as expected, with higher uncertainty in the wide likelihood condition resulting in greater estimation errors”.

In the main task, we don’t expect to see such a distinct difference in estimation errors across trial conditions because in each trial, participants are asked to combine both prior and likelihood information (that have either narrow or wide uncertainty associated) to inform their estimate of the coin’s location (i.e., the posterior). The posterior variance will always be smaller than the prior and likelihood, since the posterior precision (inverse variance) is mathematically computed as sum of the precision of the prior and the likelihood.

It is unusual that we see a main effect of prior precision on the estimation errors in the validation dataset. One reason could be the increased variability in mean estimation error in the wide prior wide likelihood condition (PwLw), in comparison to the narrow prior narrow likelihood condition (PnLn), which is particularly prevalent in the larger, validation dataset. This is visually represented in the figure below and will be included in the supplementary for readers to refer to.

**S1 Fig. Comparison of mean estimation error performance across trial conditions in A) the discovery and B) the validation datasets.** Demonstrating the variability in mean estimation errors across each of the four the trial conditions to supplement task performance analyses. Conditions: PnLn = narrow prior, narrow likelihood (red dots); PnLw = narrow prior, wide likelihood (green dots); PwLn = wide prior, narrow likelihood (teal dots); PwLw = wide prior, wide likelihood (purple dots).

*p16 - the boxes in the plots in Fig 3 are not visible, I suggest they are turned to black, or a much darker shade?*

Thank you for pointing this out. We have amended the figure as suggested (pg 16):

*p19 - Why is a Spearman correlation used in Fig 5 in the validation set but not in the training set? Also it would be helpful to plot each of the various training/validation data figures on the same axes to facilitate comparison.*

A Spearman correlation was used in both the discovery and validation datasets. This could have been clearer in text and have now included (pg 19): no significant **Spearman-ranked** correlation was found in the discovery dataset. We agree that it would aid comparison between datasets for the axes scales to be the same, so thank you for this suggestion. Figures 5 & 6 have now been updated with the same y-axis (pg 19 & pg 21) as shown below:

**Fig 5. Association between psychotic-like experiences and subjective likelihood variance.**

**Fig 6. Association between psychotic-like experiences and subjective prior variance**

*Discussion - the move from decreased subjective precision of likelihood and prior to the concept of 'task instability' is quite a big one, not really supported by evidence. As the authors say, their task is not really set up to assess subjective 'task instability' (defined as greater subjective volatility of contingencies) and in any case they don't see evidence for this in the data they have. So I don't think the authors should state in the abstract and final conclusion etc that they have found that psychotic-like experiences are associated with "greater perceived task instability".*

Thank you for this helpful comment. We agree that this is perhaps overstated in the abstract and final conclusion. We have amended a sentence in the final conclusion referring to greater perceived task instability to (pg 29-30): **Our findings suggest that psychotic-like experiences are associated with aberrant precision of encoding uncertainty in both prior and likelihood information.**

In the abstract we have similarly amended the sentence referring to greater perceived task instability to (pg 1): **Greater psychotic-like experiences were associated with aberrant precision in the encoding both prior and likelihood information, which we suggest may be related to generally heightened perceptions of task instability.**

This was also amended in the author summary (pg 2): **This was driven by the perception of greater uncertainty or unreliability associated with prior information. Psychotic-like experiences were also associated with the perception of greater uncertainty in sensory information, suggesting altered encoding of both prior and likelihood information.**

*Was there any relationship between subjective prior precision and working memory performance? Does the latter explain all the group differences in the former? One might expect that failure to remember previous trials would degrade prior precision but not likelihood precision... Also, were there any attention checks in the paradigm? If some more schizotypal participants just weren't paying much attention to the task, presumably their results would look like the obtained ones? If there weren't attention checks then it should be mentioned as a limitation.*

This is a great suggestion. We now incorporate these additional analyses in the manuscript (pg 20):

**To consider the impact of working memory on prior precision, we conducted a Spearman-ranked correlation analysis between subjective prior variance and mean spatial span (as a proxy measure of working memory performance). We found a significant, negative correlation between subjective prior variance and mean spatial span the discovery (r = -0.2, p = 2.3 x10-4) and validation dataset (r = -0.2, p = 3.0x10-8). However, we also found a significant, negative correlation between subjective likelihood variance and mean spatial span in the discovery (r = -0.17, p = 1.4x10-3) and validation dataset (r = -0.26, p = 5.1x10-13). This suggests that decreases in working memory might similarly degrade estimates of uncertainty across task in general, rather than prior precision specifically.**

While we did not include specific attention checks in the paradigm, we included quality control measures in our analyses. These points are now incorporated in the discussion (pg 28):

**Another limitation of the study is the lack of attention checks throughout the paradigm. Despite this, we included quality control measures for online testing such as removing participants with particularly low mean estimation error scores, as this was deemed to be an indicator of poor adherence or engagement with the task. Additionally, participants were asked to complete practice tasks prior to completion of the main task, to ensure that they understood what was required in the main task.**

*Last, the authors don't cite some earlier attempts to devise tasks that distinguished between prior and likelihood precision in psychiatric samples: Karvelis et al (2018, eLife) - who found no differences in subjective prior/likelihood precision in schizotypal traits (but did in autistic traits), and also Valton et al (2019, Brain) who found that Scz patients seemed to learn (visual) priors of equal precision to controls but these priors had less influence (vs those of controls) when the sensory info was weak. They could also cite Jardri et al (2017 Nat Hum Beh) who also found evidence of sensory bias over priors, and also a relation of working memory to prior weighting (but not increased likelihood variance).*

Thank you for these suggestions. We agree that these studies demonstrate valuable contributions to the literature and have included them as citations in the manuscript, including (pg 24):

**Our findings also align with previous literature demonstrating a perceptual decision-making bias towards sensory information over priors in patients with schizophrenia (Jardri et al., 2017; Valton et al., 2019).**

This is also edited in (pg 29): …this could provide an alternative explanation for the simultaneous decrease in prior and likelihood precision found in our study **and could also account for heterogeneous findings in previous literature (Karvelis et al., 2018).**
